# Food and the prison environment: a meta-ethnography of global first-hand experiences of food, meals and eating in custody

**DOI:** 10.1186/s40352-023-00222-z

**Published:** 2023-05-04

**Authors:** Clair Woods-Brown, Kate Hunt, Helen Sweeting

**Affiliations:** 1 Institute of Social Marketing and Health, University of Stirling, Stirling, FK9 4LA Scotland, UK; 2 Social and Public Health Science Unite, University of Glasgow, Glasgow, G3 7HR UK

**Keywords:** Prison, Food, Environment, Empowerment, Agency, Identity, Mental-health, Wellbeing, Cooking, Relationships

## Abstract

**Background:**

Prison foodways offer a unique opportunity to improve the physical and mental health and wellbeing of an underserved population, yet prison food is often rejected in favour of ‘junk’ food. Improved understanding of the meanings of food in prison is necessary to inform prison food policy and enhance the prison environment.

**Results:**

A meta-ethnographic synthesis of 27 papers integrated first-hand experiences of food in prison from 10 different countries. The lived experience for most in custody is of poor-quality prison-issued meals, necessarily consumed at a time and place at odds with socio-cultural norms. Beyond nutrition, food carries clear symbolic meanings in prison; through everyday food activities in prison, especially cooking, empowerment, participation, agency and identity are negotiated and performed. Cooking (with others or alone) can reduce anxiety and depression and increase feelings of self-efficacy and resilience in a socially, psychologically, and financially disadvantaged population. Integrating cooking and sharing food into the routine of prison life strengthens the skills and resources available to prisoners, empowering them as they move from the prison environment to the community.

**Conclusions:**

The potential of food to enhance the prison environment and support improvements in prisoner health and wellbeing is limited when the nutritional content is inadequate and/or where food is served and eaten impacts negatively on human dignity. Prison policy which provides opportunities for cooking and sharing food that better reflects familial and cultural identity has the potential to improve relationships, increase self-esteem, build and maintain life skills needed for reintegration.

## Background

While the primary aim of prisons is not health, it has been argued widely that prisons are in a prime position to address disproportionate health and social care issues (Baybutt et al., 2018; Brutus et al., 2012; Enggist et al., 2014). As people move in and out of prison, addressing health in prisons can also impact wider community health outcomes, reducing later costs in health care and increasing community safety (Enggist et al., 2014; WHO, 2019a). Thus, prisons represent an opportunity to tackle health problems in a way that pays a “community dividend” because any improvement in the health of the individual has a “potential knock-on effect in supporting their reintegration into community life and future health” (Stürup-Toft et al., 2018, p.3).

A key area for prisons aiming to promote health and wellbeing is enabling those in custody to develop healthy eating patterns (WHO, 2019). Providing food to prison populations, however, is a challenge which is constrained by tight budgets, complex logistics and the multiple health and social needs of the ‘customer’ (Cross, 2009; Edwards et al., 2009; Eves & Gesch, 2003). Reports of the quality of prison food vary widely from country to country. Soble et al., (2020) report on a mixed methods study conducted in prisons across 40 US states. They conclude that food in US prisons is a ‘hidden punishment’ and prisons function as food deserts which perpetuate patterns of poor health in underserved communities who already experience profound inequalities. In Spain Varoucha-Azcarate (2019) compared the diet provided in prison with national healthy eating guidance, and concluded that the meals on offer was high in fat and sugar, low in fibre and contained few fresh vegetables or fruit. An analysis of the food served in Polish prisons found that protein, fat, and carbohydrate in the diet met national recommendations but the amount of vitamins and minerals in the meals did not meet the recommended dietary allowance (Stanikowski et al., 2020). Based on results from mixed-methods studies conducted in 25 prisons across Australia, Williams et al., (2009), found that menus included adequate variety and met most nutritional standards. In the UK, Edwards et al., (2009) report on two studies conducted in 17 prisons in England and Wales which conclude that, on the whole, people in custody were provided with nutritious food.

However, in their special report of prison-issued food throughout England and Wales, Her Majesty’s Inspectorate of Prisons (H.M.I.P., 2016) determined that while “many establishments are making commendable efforts with the resources available, too often the quantity and quality of the food provided is insufficient, and the conditions in which it is served and eaten undermine respect for prisoners’ dignity” (p.13). This suggests that beyond adequate nutrition, food carries meanings in prison which can affect an individual’s personhood. Food matters in prison because interactions around food, which are characterised by top-down control and lack of choice, can augment feelings of distance from home (Comfort, 2008), impact an individual’s sense of autonomy (Smoyer & Kjaer Minke, 2015) and affect the potential of those in custody to reintegrate and care for themselves after imprisonment (Maruca et al., 2021).

A limited number of qualitative studies have been conducted on food, meals and eating in custody. However, no synthesis could be found of first-hand accounts, reported in papers globally, of how prison food impacts day-to-day prison life for those in custody. Thus, a meta-ethnography which synthesises perceptions and experiences of food, meals and eating in prison is a useful contribution to understandings internationally of the extent to which food in prisons shapes the prison environment and may be used as a tool for health and wellbeing promotion.

Meta-ethnography has emerged as one of the most well-developed methods for synthesising health-related qualitative studies (Atkins et al., 2008, Toye et al., 2013), yet details of how meta-ethnographies are conducted are often poorly reported (France et al., 2019). The method involves “systematically comparing conceptual data from primary qualitative studies to identify and develop new overarching concepts, theories, and models” (France et al., 2019, p1128). Thus, this study aims to synthesise first-hand accounts from people living in prison of eating food in prison as reported in qualitative studies globally and also add to the completeness and clarity of reporting this systematic and robust method. We chose meta-ethnography because of its emphasis on achieving new interpretive insights and meanings rather than aggregating instances (Galdas et al., 2015, Atkins et al., 2008, Erasmus, 2014, Noblit & Hare, 1988).

## Methods

Noblit and Hare (1988) outline seven phases which overlap and repeat in the development of a meta-ethnography, although they do not provide detail on how these might be achieved. Table 1 provides an overview of this process. Column A uses the same headings Noblit and Hare (1988) give to their seven stages, while column B represents the tasks we undertook after reading several meta-ethnographies (Atkins et al., 2008; Britten et al., 2002; Galdas et al., 2015; Toye et al., 2013) and literature on how meta-ethnography might be conducted and reported (Campbell et al., 2011; France et al., 2019).

**Table 1:** Seven stages of meta-ethnography with related tasks

A	B
**The seven stages of meta-ethnography**	**Tasks associated with the seven stages**
Phase 1: Getting started	Formulating a research question, a search strategy and inclusion criteria. Deciding on an approach to quality appraisal.
Phase 2: Deciding what is relevant	
Phase 3: Reading the studies	Data extraction – reading and rereading studies, establishing a coding framework. Using quality appraisal to decide on the weight given to individual studies or papers. Data analysis—coding, identifying themes, sub-themes, and relationships.
Phase 4: Determining how the studies are related	
Phase 5: Translating the studies into each other	
Phase 6: Synthesising the translations	
Phase 7: Expressing the synthesis	Reporting the process and findings of the synthesis.

### Search strategy

A comprehensive electronic search was undertaken to identify all available qualitative papers relating to the first-hand experiences of food in prison. It was based around a ‘Population, Experience, Outcome’ (PEO) structure, which has been recommended for reviews focusing on participant experiences, rather than interventions (Bettany- Saltikov & McSherry, 2016). For our research question, this was operationalised as: *population*: men and women who are in prison or who have been previously incarcerated; *experience*: food, meals (prison-issued and purchased/prepared by prisoners) and eating in custody; and *outcome*: perceived effect on identity, relationships, or the lived prison experience.

### Search processes

Search terms were formulated with the help of a specialist librarian, in relation to the PEO statement. Broad ‘population’ terms (‘prisoner’, ‘prison’,) were combined with thesaurus terms (e.g., ‘offender’, ‘inmate’, ‘jail’). This was repeated for ‘experience’ (‘food’, ‘meals’, ‘dining’). ‘Outcome’ was broken down into various aspects of prison experience (e.g., ‘identity’, ‘relationships’). Boolean operators were employed, title and abstract were included in the search parameters, the search was limited to papers written in English, and no limit was placed on publication date.

**Fig. 1:**
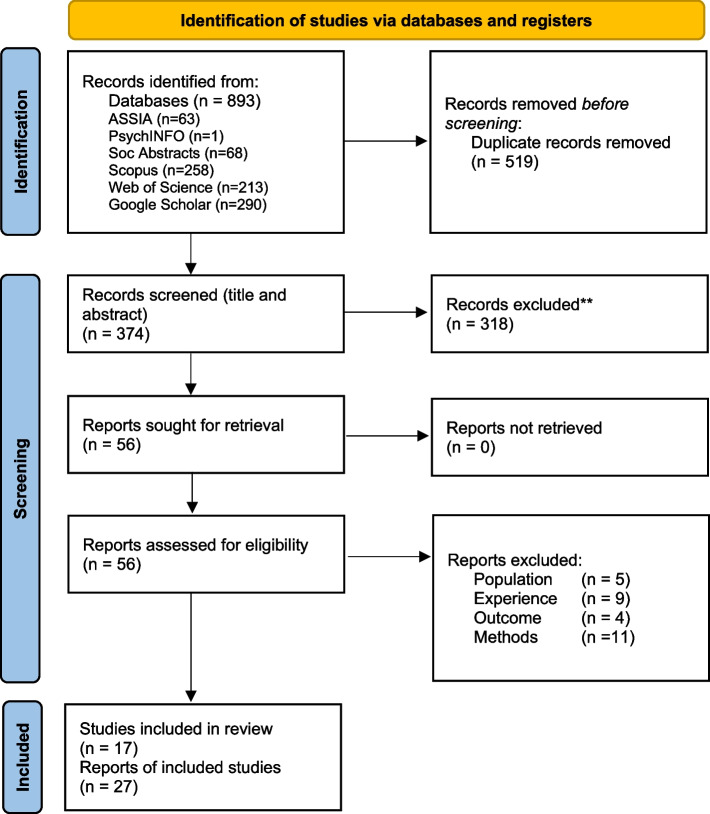
A PRISMA flow diagram (Page et al., 2021) showing records identified, screened, and included

Searches were conducted in five electronic databases (ASSIA; PsychINFO; Sociological Abstracts; Scopus; and Web of Science). Searches were conducted first in November 2019 and again in July 2022 (Fig. 1, PRISMA shows numbers for the combined searches). Google Scholar, with the broad search term ‘prison food’, was used to ensure no results had been missed by other databases. Google Scholar returned 105,000 pages (ten results per page). Full titles and abstracts were read for the first 25 pages and four further randomly selected pages (total 29 pages, 290 titles) in November 2019. In July 2022 titles were read for the first 25 pages.

### Study selection

Fifty-six papers were considered potentially eligible for inclusion because the abstracts contained two or more elements from the PEO statement; these were read in full by one reviewer (CWB). An alphabetical list of the papers was compiled, and the two co-reviewers read a random selection of these papers (HS = 15 and KH = 15). Final eligibility was based on the following inclusion criteria: *population*: participants identified as being in prison or having spent time in prison; *experience*: food, cooking, or eating in prison identified as a focus for the study; *outcome*: reports a perceived effect of prison food on identity, relationships, or the prison experience; and *methodology*: the study presents qualitative data.

The Preferred Reporting Items for Systematic Reviews and Meta Analyses (PRISMA) (Page et al., 2021) provides a template to generate a diagram which depicts the flow of information through the different phases of a systematic review. This template was adapted to report the number of records identified, included, excluded, and the reasons for exclusions (Fig. 1). Twenty-seven papers (based on 17 studies) met the inclusion criteria and are included in the review.

### Reading and data extraction approach

This is the phase where the clearest divergence between meta-ethnography and other types of qualitative evidence syntheses can be seen (France et al., 2019). France et al., (2019) suggest that details should be given of the “repeated reading of the accounts … the strategy of recording data … who was involved … and clarify which kind of primary study findings were extracted, such as first- and second-order constructs … so that readers can follow reviewers’ concept development” (p.7). Like other meta-ethnographies (Atkins et al., 2008; Campbell et al., 2011; Toye et al., 2013), we adopted Schutz’s (1976) concept of first- and second-order constructs to differentiate between the perceptions of the participants in a study (first-order construct) and the author’s interpretation of these (second-order construct) during the data extraction. The distinction between first-order and second-order constructs is not always clear (Atkins et al., 2008), especially when dealing with multiple papers, thus Toye et al., (2013) suggest interpretations of first- and second-order constructs are “negotiated and constructed collaboratively” (Toye et al., 2013 p.13).

CWB and HS independently identified first- and second-order constructs in two randomly selected papers (data were extracted from all sections of all papers). Interpretations of first- and second-order concepts were compared, and it was agreed that quotes from those in custody would be extracted as first-order constructs and interpretations developed by authors as second-order constructs. CWB then extracted all first- and second-order constructs from all papers. HS did the same for a random selection of 10 papers. CWB and HS met once again to discuss the data extraction process and compare their approaches.

**Fig. 2:**
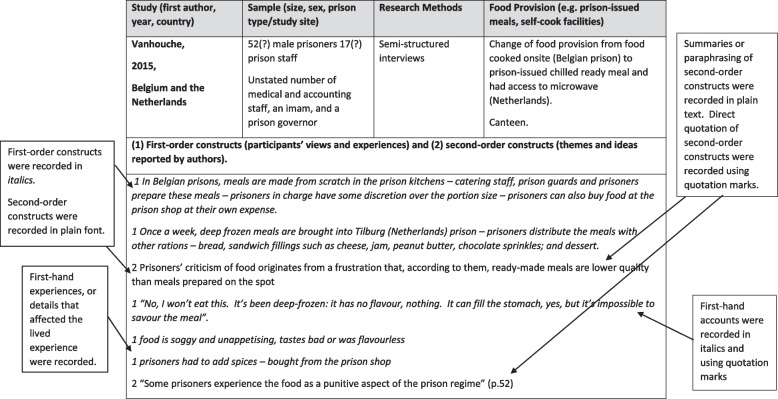
An extract from the data table showing how first- and second-order constructs were recorded

Overall, there was agreement, but CWB raised questions about the status of descriptions of material aspects of prison food found in the papers, for example around the timing of meals in prison, or where or how food was served. While not direct quotes, these details were important to building up a picture of everyday experiences around food in prison. It was agreed to extract these details as first-order constructs. Finally, a data table (Fig. 2) was produced by CWB using Word which recorded all first- and second-order constructs from the 27 included papers. This table also set out characteristics of the studies, including, year of publication, related papers, country of study, population, number of participants, prison type/study site, type of food provision detailed, and data collection methods used. This data table was cross-checked for accuracy by HS and KH.

### Quality appraisal strategy

Alongside the data extraction process, we undertook quality appraisal. As Britten et al., (2002) note, it was more efficient to do these two activities at the same time, as they both required detailed reading of all the papers. The position we adopted to quality appraisal followed Galdas et al., (2015), where the process was used to “provide information on the quality of included studies rather than as a basis for inclusion” (p.46). Quality appraisal took place after screening, and focused on balancing insights gained from any one study with any shortcomings there may have been in methodology (or the reporting of this) in order to determine a study’s place in the final review (Luciani et al., 2020).

Elements from the Critical Appraisal Skills Programme (CASP, 2018) checklist and Spencer et al., (2003) framework for assessing qualitative studies were used in designing a quality appraisal tool. These were useful in framing questions around the research design, findings and reporting of studies. In addition, note was taken of Franzel et al., (2013) who recommend ( +) or (-) as a straightforward way to indicate how well a particular aspect of research design, findings or reporting is covered by an individual paper. They also suggest this is a simple way to assist with decisions about the weighting a study or paper is given in the final synthesis. Designing the quality appraisal tool was also informed by Gough (2007) who emphasises the need to pay attention to the context of any study when deciding its place in the final synthesis. Thus, a study may have accrued a number of ( +) in terms of research design, findings, or reporting; however, the cultural context, setting or sample of the study may have been so idiosyncratic that the study’s overall weighting in the final synthesis is reduced. The final design of the quality appraisal tool was agreed by all reviewers (Table 2).

**Table 2:** Quality appraisal tool designed and used to assess the quality of included papers

Quality Appraisal Tool To be completed for each individual paper			
Reference:		±	Context (setting/sample):
Research Design	Are the research questions clear?		Comment:
	Is there a clear statement of methodology?		
	Is sampling clearly described?		
	Is data collection clearly described?		
	Is there justification for methodology?		
	Are limitations acknowledged and discussed?		
Findings	Are the findings credible?		Comment:
	Are claims made supported by sufficient evidence?		
Reporting	Is the method of data analysis clearly described?		Comment:
	Are the data, interpretation, and conclusions well documented?		
	Is the paper clear, coherent, and well structured?		
Does the study make a useful contribution to knowledge and understanding of experiences of food, meals and eating in the prison context?		Comment:	
Does the cultural context of the setting/sample affect the weighting given to the study?		Comment:	
Overall quality appraisal rating		LowModerateHigh	

Using the quality appraisal tool one reviewer (CWB) appraised all 27 papers, the other two reviewers appraised several randomly assigned papers (HS = 17 and KH = 10). Reviewers independently read and completed the appraisal tool checklist for each paper and met to discuss the overall weighting (low, moderate, or high) that a paper would be given in the final synthesis. We agreed on the overall quality appraisal and final weighting for most studies (*n* = 14/17). Where there was disagreement, discussions centred around the reporting of methodology and limited evidence for claims or conclusions. Consensus was reached after rereading papers and reemploying the quality appraisal tool together. The final QA judgement assessed six of the 27 papers as ‘high’, 17 as ‘moderate’ and four as ‘low’ quality for this review (Table 3).

**Table 3:** Final quality appraisal decisions on included studies and related papers

^Quality Appraisal^ ^Questions^	Research Design						Findings		Reporting			Place in final synthesis			
^Study^	Clear RQs	Methodology	Sampling	Data Collection	Justification of Methods	Limitations	Credible findings	Well evidence claims	Methods of analysis	Conclusions	Clear and coherent	Useful Contribution	Context affects weighting	Weighting of individual paper/study	Overall weighting of study with multiple papers
Blore, 2011															
Chatterjee and Chatterjee 2018	-	+	+	+	+	+	+	+	-	+	+	+	Y	M	
Graaf and Kilty 2016	+	+	+	+	-	+	+	+	-	+	+	+	Y	M	
Earle, 2012	+	+	-	-	+	+	+	+	-	+	+	+	N	H	
Einat, 2018	+	+	+	+	+	+	+	+	+	+	+	+	Y	M	
Godderis (2006a)	-	+	+	+	+	-	+	+	-	+	+	+	Y	M	M
Godderis (2006b)	-	+	+	+	+	-	+	+	-	+	+	+	Y	M	
Ifeonu, 2022	+	+	+	+	+	-	+	+	-	+	+	+	Y	M	
Hannan-Jones, 2016	+	+	+	+	+	+	+	+	+	+	+	+	Y	M	
Heckenberg, 2006	-	-	-	-	-	-	+	+	-	-	+	+	N	L	
Kjaer Minke, 2014	+	+	+	+	+	-	+	+	+	+	+	+	N	H	M
Smoyer, 2019	-	+	+	+	-	+	+	-	+	-	+	+	Y	L	
Parsons, 2017	+	-	-	-	-	+	+	+	-	+	+	+	Y	M	H
Parsons, 2018	+	+	+	+	+	-	+	+	+	+	+	+	N	H	
Parsons, 2020	+	+	+	+	+	-	+	+	+	+	+	+	N	H	
Smith, 2002	+	+	+	+	+	+	+	+	+	+	+	+	N	H	
Smoyer, 2013	+	+	+	+	+	+	+	+	+	+	+	+	Y	M	
Smoyer, 2014	+	+	+	+	-	+	+	+	-	+	+	+	Y	M	
Smoyer (2015a)	+	+	+	+	-	+	+	+	+	+	+	+	Y	M	
Smoyer (2015b)	+	+	+	+	-	-	+	-	+	-	+	+	Y	L	M
Smoyer, 2016	+	+	+	+	-	+	+	+	+	+	+	+	Y	M	
Smoyer, 2017	+	+	+	+	-	+	+	+	+	-	+	+	Y	M	
Ugelvik, 2011	-	-	-	-	-	-	+	+	-	-	+	+	Y	L	
Valentine, 1998	-	+	+	+	-	+	+	+	-	+	+	+	N	H	
Vanhouche, 2015	+	+	-	+	+	-	+	+	+	+	+	+	Y	M	M
Vanhouche, et al., 2018	-	+	+	+	-	+	+	+	-	+	+	+	Y	M	
Williams et al., 2008	+	+	+	+	+	+	+	+	+	+	+	+	Y	M	

One was considered ‘low’ quality because of its sample (six former prisoners) (Heckenberg & Cody, 2006), two because of limitations in providing evidence for conclusions (Smoyer, 2015a; Smoyer & Minke, 2019) and the other because there were insufficient details about research design and data analysis to judge the study’s rigour (Ugelvik, 2011). These papers were given less weight in the final synthesis (although first-order constructs from these papers were included if they offered useful insights). Differences between high and moderate quality papers were judged not significant enough to warrant differentiation in the final synthesis.

### Determining how studies are related

In meta-ethnography, first-order constructs (participants words/experiences) and second-order constructs (authors’ interpretations of participants words/experiences) are further abstracted into third-order constructs (Schutz, 1976). Third-order constructs are reviewers’ “interpretations of the original authors’ interpretations of participants’ interpretations of their experiences” (Malpass et al., 2009, p.158). Noblit and Hare (1988) advise that this phase of meta-ethnography involves finding meaning across all the “key metaphors, phrases, ideas and/or concepts” (p.28) identified in the data. Toye et al., (2013) note that this process is iterative; as the reviewer reads across studies, they “have to recode findings or to condense them into higher conceptual categories to make sense of them” (p.12).

**Fig. 3:**
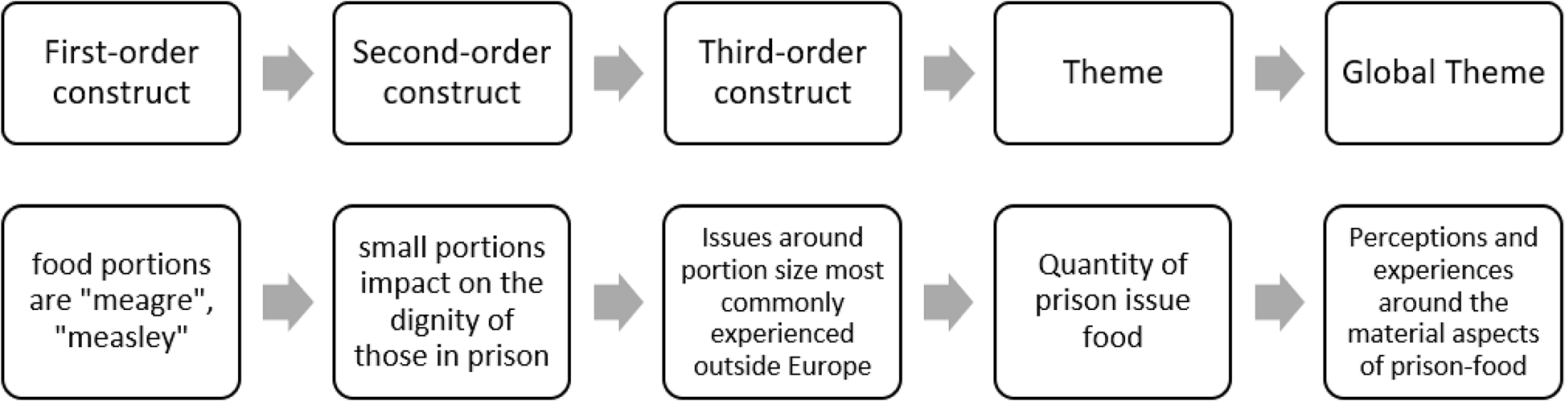
The process of abstraction from first-order construct to global theme

For this phase of the meta-ethnography two reviewers (CWB and HS) read and reread the data table. As we read, we made note of commonalities between first- and second-order constructs, thus identifying emerging third-order constructs independently. All reviewers met to discuss how third-order constructs might be grouped, merged, and organised into themes. Figure 3 shows an example of how a first- and second-order construct was abstracted into a third-order construct, then into a theme and as a part of a global theme.

Following this discussion, CWB constructed a thematic framework of themes and overarching global themes. This was discussed with HS and KH and adjustments were made to terms used to ensure clarity and accuracy and a final thematic framework was produced (Table 4).

**Table 4:** Organisation of thematic heading and global themes

**Global Theme One: Perceptions and experiences around the material aspects of prison food**				
Quality of food	Quantity of food	When and where food was eaten		Food and prison routine
**Global Theme Two: Experiences of prison foodways as punishment, power, resistance and agency**				
Punishment	Power	Resistance		Agency
**Global Theme Three: Prison foodways, identity, self and relationships**				
Cultural identity	Self		Relationships	

### Translating studies into each other

A key aspect to any meta-ethnography is what Noblit and Hare (1988) term “translating one study into another” (p.28). However, as Atkins et al., (2008) observe, there is a lack of clarity in their original text as to how this might be done. We interpreted this phase as a process of understanding how the first- and second-order constructs across studies were alike and not alike and how they might be arranged within the thematic framework, i.e., do the concepts of the studies say similar things about the theme? CWB created a Word document for each global theme with themes as sub-headings. First- and second-order constructs which said similar things were grouped together and outliers highlighted, third-order constructs were added to each grouping. The findings of the quality appraisal were considered in this phase too, with those studies that scored ‘low’ also highlighted in the Word document. These three documents were shared with the other reviewers, checked for accuracy and the type of synthesis we might produce discussed.

### Synthesising translations

The final phase in the meta-ethnography involves “making a whole into something more than the parts alone imply” (Noblit & Hare, 1988, p.28). Erasmus (2014) notes that this means creating a “coherent and integrated articulation of the accounts presented in the individual papers; a statement that transcend[s] the knowledge statements or claims of any of the individual papers” (Erasmus, 2014, p.6). Noblit and Hare (1988) suggest there are three ways in which a synthesis might be articulated. These are: (1) refutational (in which findings contradict each other); (2) reciprocal (in which findings are directly comparable); and (3) findings are taken together and interpreted as a “line of argument” (p.28). The studies we identified present common themes, and all illuminate the same thing, i.e., experiences of prison food; while comparable, the contexts of the studies vary widely. Thus, we decided a line of argument would best articulate our findings.

**Fig. 4:**
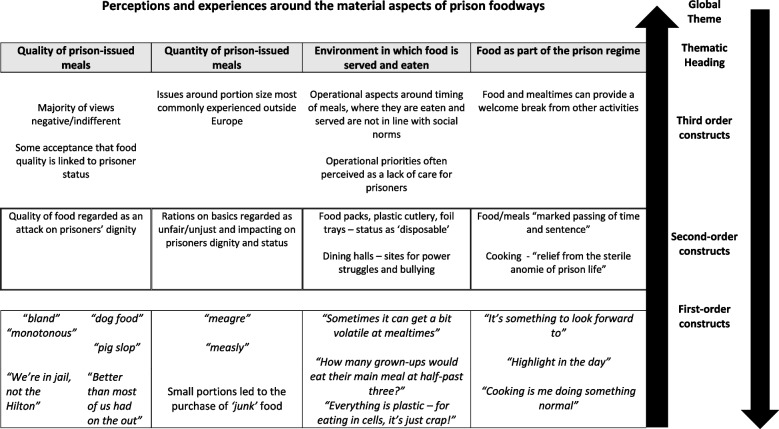
Thematic Hierarchy One: Perceptions and experiences around the material aspects of prison food

**Fig. 5:**
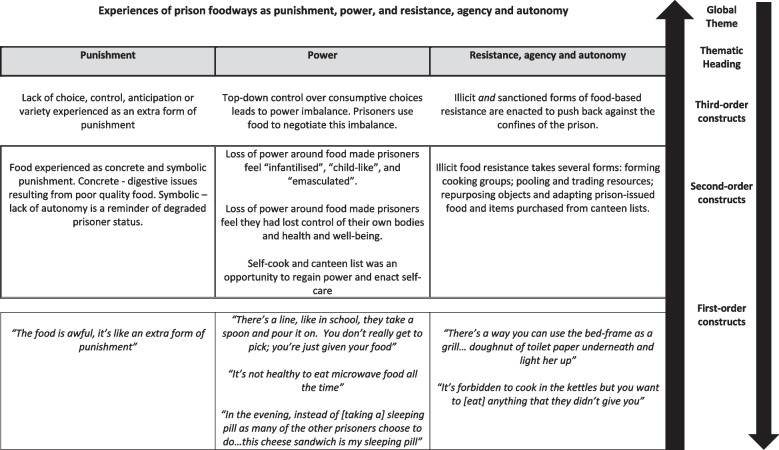
Thematic Hierarchy Two: Experiences of prison food as punishment, power, resistance, agency and autonomy

**Fig. 6:**
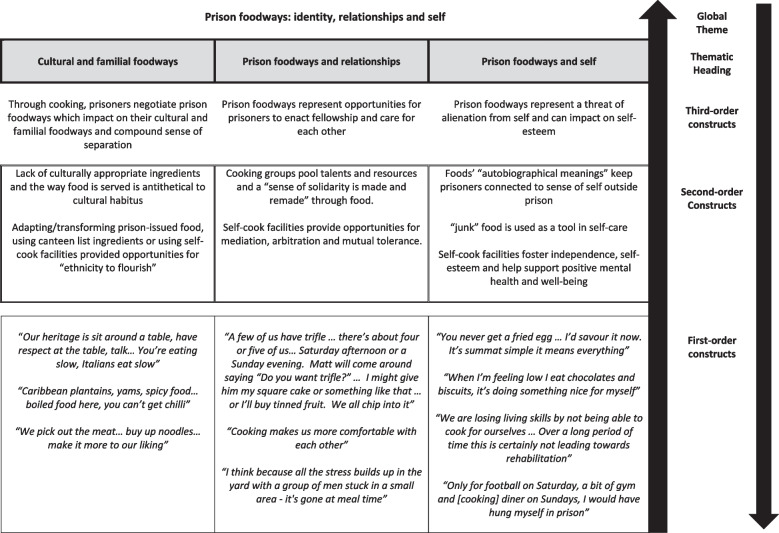
Thematic Hierarchy Three: Prison Food - identity, relationships, and self

At the start of the synthesis process, CWB and HS used the three Word documents to construct thematic hierarchies which gave an overview of global themes. Three thematic hierarchies were created (Figs. 4, 5 and 6). These were shared with KH who checked to ensure they represented the data clearly and accurately. Taken together, these thematic hierarchies represent new conceptual frameworks around first-hand experiences of food in prison and help articulate the line of argument that: *Experiences around food in prison, where choice and control are limited, affect prisoners’ lived experience of prison and their perceptions of status, identity, and social relationships.*

Our line of argument synthesis integrates first-hand experience of food in prison from 17 studies, reported in 27 papers globally. The synthesis takes account of experiences in 10 different countries; the prison food experiences of both men and women in custody; and different food provision, including prison-issued meals, self-cook facilities and prison canteen (a shop from which those in custody can buy from a set list of products including food items, usually delivered once per week). Table 5 provides an overview of the study characteristics.

**Table 5:** An overview of included study characteristics (*studies are numbered 1–27)

Study/Paper: first author, year,	Country	Sample (size, sex, prison type/study site)	Food Provision/s
Earle and Phillips 2012^1*^	UK, England	50 men in medium security prison	Self-cook
Parsons, 2017^2^, 2018^3^, 2020^4^	UK, England	Unstated number of men on day release at a resettlement scheme.Men in category C prison	Café kitchen/dining room of day release programme Prison issued meals, prison shop
Smith, 2002^5^	UK, England	89 women across three prisons; one closed, one open and one remand prison	Prison-issued meals, self-cook in open prison, prison shop
Valentine and Longstaff 1998^6^	UK, England	Unstated number of men	Prison-issued meals, prison shop
Blore, 2011^7^	Australia	Sample of prisoners from Prisoner Advisory Committees in State of Queensland. Number of participants not stated	Prison-issued meals, prison shop
Hannan-Jones, 2016^8^	Australia	120 male prisoners, unstated number of food service staff in a maximum-security prison	Prison-issued meals
Heckenberg and Cody 2006^9^	Australia	Six men previously incarcerated males (all had been resident in the maximum and medium security wings of the same prison)	Prison-issued meals
Williams et al., 2008^10^	Australia	27 men and nine women. Three prisons, one male maximum security, one male minimum security, and one female prison	Prison-issued meals, prison shop
Graaf and Kilty 2016^11^	Canada	12 previously incarcerated women resident in a transition house	Prison-issued meals, prison shop
Godderis, 2006a^,12^, 2006b^,13^	Canada	17 men across three prisons, two medium-security and one minimum-security	Prison-issued meals
Ifeonu et al., 2022^14^	Canada	495 men and 92 women in four prisons	Prison-issued meals, prison shop
Kjaere Minke, 2014^15^	Denmark	68 men in a maximum-security prison	Self-cook
Smoyer, 2019^17^	Denmark	9 women across remand centre, closed prison and open prison	Self-cook
Ugelvik, 2011^17^	Norway	Unstated number men resident in two remand wings in one prison	Prison-issued meals, prison shop
Vanhouche, 2015^18^, 2018^19^	Belgium and Netherlands	52 men transferred from a Belgian prison to a Dutch one, 17 prison staff and unstated number of medical and accounting staff, an imam, and a prison governor	Prison-issued chilled ready meal access to microwave. prison shop
Smoyer, 2013^20^, 2014^,21^, 2015a^,22^, 2015b^23^, 2017^24^, 2017^25^	USA	30 previously incarcerated women resident in a transition house	Prison-issued meals prison shop
Chatterjee and Chatterjee 2018^27^	India	90 women across two prisons, one mixed, one female only	Prison-issued meals, prison shop
Einat, 2018^27^	Israel	20 previously incarcerated men resident in a transition house	Prison-issued meals, prison shop

### Synthesis findings

#### Global theme one—perceptions and experiences around the material aspects of prison food

This global theme draws together those aspects of food, meals, and eating in prison that were most frequently reported by people in custody (first-order; *italicised*) and by the author(s) interpreting these experiences (second-order; plain text).

#### Quality of food received

Negative perceptions and experiences of prison-issued food were reported across all 10 countries. Some study participants regarded the food as *‘terrible’* and ‘*disgusting’* (Blore, 2011, p.89) and likened it to ‘*animal grade’ ‘dog food’* or *‘pig slop*’ (Graaf and Kilty 2016, p.32; Heckenberg and Cody, 2006, p.18; Smoyer, p.17), while others were indifferent to prison-issued food and experienced it as simply bland or monotonous (Britten et al., 2002; Brutus et al., 2012; Collica 2010). There were complaints from participants in several studies about the lack of fresh fruit and vegetables in their diets (Brutus et al., 2012; CASP 2018; Chatterjee and Chatterjee 2018; Comfort 2008; Cross et al., 2009; Dooris et al., 2014; Enggist et al., 2014; Godderis 2006a; Graaf and Kilty 2016).

None of the studies reported any positive comments about the quality of prison-issued food. However, some noted acceptance that any food, whether good or bad, would be poorly received by people in custody *because* they were in prison, *‘At the end of the day, steak or Spam, prison food is prison food’* (first-order, Smith, 2002, p.204); ‘*We’re in jail, not the Hilton’* (Vanhouche, 2015, p.51); ‘It’s prison. What the f*** do they (other prisoners) expect (Ifeonu et al., 2022) and ‘*Can the food be good here? This is after all a jail’* (Chatterjee and Chatterjee, 2018, p.51). There was also some recognition among some of those in custody that prison provided regular access to meals that was lacking in their lives in the community, characterised by poverty, unstable housing and illicit drug use (Blore 2011; Collica 2010; Earle and Phillips 2012; Franzel et al., 2013; Graaf and Kilty 2016).

What we could not glean from the data is whether the majority of prison-issued food in the various prisons studied *is* of poor quality. However, whether because of the lack of choice or control around prison food, or the quality of the food itself, most people in custody represented in this meta-ethnography perceived and experienced the quality of prison-issued food negatively. This was constructed as problematic by study authors because it led to rejection of prison-issued food in favour of unhealthy snacks from the canteen (Blore 2011; Campbell et al., 2011; Chatterjee and Chatterjee 2018; Collica 2010; Franzel et al., 2013).

#### Quantity of Prison-issued Food

Eleven out of the 17 studies reported on the quantity of prison-issued meals. Portion sizes were described as *‘measly’, ‘meagre’*, and ‘*insufficient’* (first-order in Blore, 2011; Smoyer, 2013; Vanhouche, 2015). Issues around portion size were most often reported in studies outside Europe (especially in Canada and the US), where those in custody complained about being constantly hungry (Collica 2010; Cross et al., 2009; Edwards et al., 2009; Eves and Gesch 2003; Graaf and Kilty 2016). Only two of the seven European studies mentioned portion size (Britten et al., 2002; Dooris et al., 2014). In studies outside Europe, experiences around insufficient portion size, especially of basics such as bread and cereals, were equated with unfair treatment, a lack of dignity, and a loss of status (Brutus et al., 2012; Campbell et al., 2011; Collica 2010; Comfort 2008; Enggist et al., 2014; Godderis 2006a; Graaf and Kilty 2016). This was especially true among those who did not have sufficient funds with which to augment their diet from the canteen (Campbell et al., 2011; Comfort 2008; Enggist et al., 2014). Like the perceived quality of the food, negative experiences around portion size led to ‘filling up’ on purchases of ‘junk’ from the canteen (Campbell et al., 2011; Collica 2010; Comfort 2002; Eves and Gesch 2003; Franzel et al., 2013; Graaf and Kilty 2016).*‘To actually feel full around here, you actually have to get canteen’*(Ifeonu et al., 2022).

#### When and where food and meals were served and eaten

This issue was raised in eight of the moderate/higher quality studies. These included seven from the 10 conducted outside Europe (Brutus et al., 2012; Chatterjee and Chatterjee 2018; Collica 2010; Comfort 2008; Enggist et al., 2014; Franzel et al., 2013; Godderis 2006a) and one from England (Britten et al., 2002). Concerns were marked by resentment at the lack of control and choice around the timing of prison-issued meals, as reported in two Australian studies: ‘*You get fed at three thirty in the afternoon, you have to [eat it then] because it’s hot*’ (Williams et al., 2008 p.10); ‘*How many grown-ups eat their meal at half-past three … It’s not normal’* (Heckenberg and Cody, 2006, p.10). People in custody also resented the long gap between the evening meal and breakfast, which could extend to 17 h on weekdays and longer at weekends (Britten et al., 2002; CASP 2018; Chatterjee and Chatterjee 2018; Enggist et al., 2014).

Parsons (2020) suggests that operational decisions, such as issuing food packs containing items for breakfast or lunch to be eaten in cells, could be interpreted by those in custody as a lack of care or concern by the institution. This is echoed in several other studies where prepackaged convenience food was associated with indifference on the part of the prison authorities (Bettany- Saltikov and McSherry 2016; Collica 2010; Dooris et al., 2014; Earle and Phillips 2012) and underlines the “lowly status associated with being a prisoner” (Parsons, 2020, p.4). Smoyer (2013) asserts that such operational decisions caused “the human experience of eating [to be] transformed into an unpleasant act which was nonhuman, mechanical, or animal-like, devoid of social interaction” (second-order, p.117).

Dining halls, could be a site for tension, precarity and intimidation. Godderis (2006a) cites one respondent who described the dining hall as ‘volatile’ (p.274) and an important area for displays of power, and the marking of territory where ‘you want to make a name for yourself’ (p.274). These experiences are echoed by Valentine and Longstaff (1998), who observed that in dining halls, weaker men were intimidated into giving their food away, and by Blore (2011) who observed “power struggles” (p.90) in the dining hall.

#### Food and the prison routine

As Valentine and Longstaff (1998) state, food related activities can “mark the passing of time in both senses of the word: day and sentence” (p.134). Despite the tensions outlined above, people in custody in several studies reported that mealtimes were one of the most significant aspects of prison life; they offered a welcome break from other activities and helped structure the monotonous and uniform prison day (Atkins et al., 2008; Britten et al., 2002; Campbell et al., 2011; Chatterjee and Chatterjee 2018; Collica 2010; Enggist et al., 2014; Godderis 2006a). The capacity of prison foodways to afford respite from the tedium of the prison regime was magnified in the studies of prisons where there was access to cooking facilities (Atkins et al., 2008; Blore 2011; Cross et al., 2009; Earle and Phillips 2012; Godderis 2006a).

#### Global theme two – experiences of prison food as punishment, power, resistance, agency and autonomy

##### Punishment and Power

De Graaf and Kilty (2016) suggest that how individuals understand their treatment in custody is mediated through the food they receive; the quality, quantity, and timing of prison-issued meals, along with where these meals are served, act as part of the disciplinary and control process (Collica 2010; Comfort 2008; Enggist et al., 2014). Smith (2002) includes a quote from one respondent who expresses this explicitly, ‘*It’s like being on punishment here … the food is awful, it’s like an extra form of punishment’* (p.202). Some studies suggest that through the prison-issued food itself, and the manner in which it is served and eaten, food is experienced as both a concrete *and* symbolic form of punishment (Dooris et al., 2014; Enggist et al., 2014; Graaf and Kilty 2016).

Perceiving or experiencing prison food as punishment is widespread across the studies and the countries represented. In the UK, the lack of autonomy, variety and excitement around food was regarded as punitive (Blore 2011; Britten et al., 2002). In Denmark, the prison-issued food was seen as particularly punitive by immigrants who reported finding it hard to digest (Dooris et al., 2014). In Vanhouche’s (2015) study, the move from a prison where food was cooked and served directly from the kitchen (in a Belgian prison) to pre-packaged microwave meals (in a Dutch prison) was experienced as a punitive part of the new prison regime. Digestive issues resulting from the prison-issued food and the lack of fresh produce was experienced as an extra form of punishment by prisoners in Australia, Canada and the US (Chatterjee and Chatterjee 2018; Collica 2010; Enggist et al., 2014). In India and Israel, prison food was regarded as an extension of a cruel and uncaring ‘state’ (Franzel et al., 2013; Godderis 2006a).

Most of the studies (*n* = 14/17) report on food systems where those in custody have little or no control over their food consumption. This was framed by authors as indicative of a power imbalance (Britten et al., 2002; Brutus et al., 2012; Campbell et al., 2011; Chatterjee and Chatterjee 2018; Collica 2010; Comfort 2008; Franzel et al., 2013) which infantilised, *‘There’s a line, like in school. You don’t really get to pick,you’re just given your food’* (Smoyer, 2013, p.120). This is echoed by Ugelvik (2011); “The official food positions the prisoners as emasculated and child-like” (p.54).

In contrast, Smoyer and Minke (2019) report that the Danish self-cook facility affords prisoners the opportunity to take care of their mental health and wellbeing by making ‘hygge’ (a Danish word which refers to creating a cosy atmosphere and sense of wellbeing), *‘Instead of [taking a] sleeping pill as many of the other prisoners choose to do…this cheese sandwich is my sleeping pill … it's no use just to cry. You have to make hygge in your cell’* (p.3). Cooking one’s own food in this instance is regarded as an alternative to medication and a way to regain power around self-care. Earle and Phillips (2012) suggest that in preparing and sharing food the prisoners in their study “found relief from the sterile anomie of prison life … [to] transcend the dehumanising and mortifying conditions of their incarceration" (p.145). Similarly, Smith (2002) asserts that where prisoners are permitted to cook for themselves, they experience it as an "immense pleasure" which they regard as "humanising" (p.202).

##### Resistance, agency and autonomy

Overt food-based resistance is not widely reported in the studies and was largely confined to ‘kicking off’ (Smith, 2002, p.205) in the dining hall (Collica 2010; Comfort 2002; Comfort 2008). Widely reported across the studies, however, is illicit food-based resistance, agency and autonomy. There are several descriptions of those in custody taking control of their own consumptive choices by adapting or repurposing prison-issued food in creative, sometimes dangerous, ways: *‘Someone’ll save a cold burger … And some lads try cooking in the cell, they get a tray light a fire … with a bit of butter and cook it up’* (Valentine and Longstaff, 1998, p.141). Godderis (2006b) reports on an individual making a grill by balancing their bedframe on books and burning toilet paper. Vanhouche (2015) explains that the lack of cooking facilities in Belgian prisons led individuals to make their own, involving small fires in cells: *‘We have to be creative in the way we cook … Like deep-frying … we put oil in a saucepan and fry … in fact it is dangerous’* (p.51). Thus, people in custody take risks to enact agency and autonomy over their consumptive choices. Some suggest that illicit cooking is a means of surviving in a hostile environment, *‘It’s not that I want to break any rules … I just want to survive. You have to do something to survive in this place’* (Ugelvik, 2011, p.51). Parsons (2020) reports on individuals pooling their resources to “recreate memories” and counter the “alienating impact” (p.4) of the prison environment. Across the studies it is suggested dishes constructed collaboratively help prisoners resist the pains of imprisonment (Baybutt et al., 2014; Bettany- Saltikov and McSherry 2016; Blore 2011; Collica 2010; Dooris et al., 2014; Dooris et al., 2014) and allow those in custody to “push back against the system that confined them and the sense of powerlessness that it produced” (Smoyer, 2013, p.130).

#### Global theme three – prison food: identity, relationships, and self

##### Cultural and Familial Food

Blandness and a lack of culturally appropriate foods were highlighted in several studies. Two English studies (Atkins et al., 2008; Britten et al., 2002), conducted 14 years apart, describe similar issues. In the first, an individual highlights the differences between his diet outside and inside prison: ‘*[Outside] I would eat more Caribbean vegetables and fruit … more spicy food, whereas here it’s more like boiled food, here you can’t get chilli*’ (Valentine and Longstaff, 1998, p.136). In the second, 14 years later, little has changed: *‘Black guys probably find the food they serve … alien or disgusting … [to] the average Jamaican … it’s really bland … It’s like going to a [highway] service station 30 years ago’* (Earle and Phillips, 2012, p.150).

Criticisms about the bland nature of prison food recurred frequently. Across the data the official food is transformed by people in custody into something that they believe tastes more like home and reflects their cultural identity. In Australia, an Asian individual described his practice*, ‘I’ll just pick the meat out … buy-up some noodles and I’ll put this in with the noodles and make it taste a bit more to our liking’* (Williams et al., 2008, p.9). In another Australian study (Hannan-Jones and Capra, 2016), a Turkish individual described secretly culturing yoghurt for months using prison-issued milk and a culture from a yoghurt he had been given as part of his breakfast pack. In Canada, Ifeonu et al., (2022) report an account of one individual making toffee in prison *‘He gets a bunch of peanut butter, a bunch of honey, and a bunch of sugar, and he makes his own toffee, right. He did it all up, melted it, and put it in the fridge. He went around and sold chunks’*. In Ugelvik’s study (2011), Eastern European prisoners in Oslo illicitly made cheese from the daily milk they were given.

Those in prison who demonstrate culinary knowledge can use this as social and cultural capital to build supportive relationships, access additional resources or improve their standing among other prisoners. Earle and Phillips (2012) describe ‘boats’ (groups of prisoners), as “complex and intimidating hierarchies for the distribution of labour, skills and resources around food” (p.147). Men gained social and cultural capital for their abilities in the kitchen, with a Turkish baker able to rise above inherent racial tensions because of his skills in baking and icing elaborate birthday cakes. Other men were celebrated because they knew how to use spices which “operated as informal currency” (Earle and Phillips, 2010, p.147). Kjaer Minke (2014) suggests that those with cooking skills were able to dictate the terms on which cooking groups were formed. While other prisoners in food groups pooled their food resources, the cooks traded with their cooking skills, taking others’ ingredients, and producing elaborate dishes for the group.

In some studies, the manner in which food is served is regarded as “antithetical” (Smoyer, 2013, p.182) to maintaining cultural identity. Those interviewed in Smoyer’s (2013) study complained about the prison guard hurrying them at mealtimes, *‘Our heritage is to sit around the table … talk together … Italians eat slow’* (p.182). Parsons (2020) also suggests that the way food is eaten in prison is perceived to alienate individuals from their cultural habitus:

*You don’t eat with metal knives and forks … Everything is plastic. You don’t sit down … everybody is back in their cells, eating off their own lap … which is pretty crap really … my mother was always one for eating at the table … we tended to do that at home as well (p.4).*

De Graaf and Kilty (2016), meanwhile, suggest that the safety measures taken by giving prisoners plastic cutlery are perceived as demeaning—“throwaway utensils for a throwaway population” (p.34).

##### Prison food and Relationships

Through pooling resources and cooking and eating together, fellowship, support and care is enacted among those in custody. Members of cooking groups together created more elaborate dishes than skills or resources would otherwise allow. Parsons (2020) reports an example of this, ‘*There’s about four or five of us … Matt … say[s] “Do you want trifle?” … I might give him my square cake … or I’ll buy tinned fruit. We all chip into it*’ (p.6). Food groups, whether constructed for the illicit cooking and sharing of meals (Chatterjee and Chatterjee 2018; Comfort 2008; Dooris et al., 2014; Dooris et al., 2014; Enggist et al., 2014) or formed in prisons with some self-cook facilities (Atkins et al., 2008; Bettany- Saltikov and McSherry 2016; Blore 2011; Cross et al., 2009), demonstrate that “Through preparation of meals for [and with]others, a sense of solidarity or sociability is made and remade” (Parsons, 2020, p.6). One individual in Earle and Phillips’ (2012) study said the self-cook facility ‘*made people more comfortable with each other*’ (p.147). In her study of a self-cook facility in Denmark, Kjaer Minke (2014) found similar positive relations between prisoners in food groups, *‘If we sit and eat together on a daily basis … I would stand by you if you’re having problems’* (p.233).

##### Prison food and the self

Smith (2002) suggests that food is a key tool in handling the stress and anxiety of prison, acting as an “important self-help mechanism, representing the construction and maintenance of a viable sense of self-control” (p.206). She reports that in her study, those who engaged in “risky” or “unhealthy” food-related behaviours, did so as a means of coping with their loss of control, ‘*When I’m feeling low, I eat chocolates and biscuits because it’s doing something nice for myself. I don’t mean nice in the sense that it’s healthy, but that I like doing it’* (p.204). Chatterjee and Chatterjee (2018) report similar findings from their study of women in Indian prisons; individuals ate biscuits, chocolate and fries to “escape the perpetual control prison has over their day to day lives” (p.32). The resultant negative effects on health were regarded as “worth the trade off in gustatory pleasure” (p.52).

Godderis (2006b) suggests that the lack of choice and control prisoners felt around prison foodways resulted in a “sense of alienation from their physical selves” (p.68). Half the studies (*n* = 8/17) report on the effect that prison foodways can have on individuals’ perception of their sense of self. For some prisoners it was the lack of choice around food that emphasised they had lost the right to make decisions about their own lives, ‘*This is why it’s become so hard for me in here because I’m always given [food], got no choice in the matter’* (Valentine and Longstaff, 1998, p.135). Frustration at the lack of choice around food and how this negatively affected the self-esteem of those in custody is noted in several studies (Blore 2011; Brutus et al., 2012; CASP 2018; Chatterjee and Chatterjee 2018; Comfort 2008; Enggist et al., 2014; Godderis 2006a). A number of authors suggest that individuals’ inability to make decisions about what, where and when they ate, was a constant reminder of their degraded status as prisoners (Blore 2011; Collica 2010; Comfort 2008; Dooris et al., 2014; Enggist et al., 2014; Franzel et al., 2013; Graaf and Kilty 2016).

In contrast, it seems that those who had more choice and control around food, meals, and eating regarded food, particularly cooking, as a lifeline that kept them going in difficult circumstances, *‘Only for football on Saturday and a bit of gym and [cooking] dinner on Sunday, I would have hung myself in this prison’* (Earle and Phillips, 2012, p.146). Parsons (2017), in her study of the café and kitchen of a day release and resettlement facility, suggests that cooking lunch for themselves and others increased the self-esteem, self-respect, and self-confidence of those in custody, ‘*It enables you to have conversations … to define who you are a bit’* (p1086). Ifeonu et al., (2022) suggests that as food is acquired, exchanged, prepared, and consumed, in prison it becomes invested with a value that speaks to a person’s standing in the prison community and that food-related identities are used as forms of distinction.

Evidence from several studies suggests food is also capable of connecting those in custody with both their sense of who they were outside prison and their families (Atkins et al., 2008; Brutus et al., 2012; Chatterjee and Chatterjee 2018; Collica 2010; Comfort 2008; Dooris et al., 2014; Enggist et al., 2014; Godderis 2006a):

*Sometimes I just close my eyes and remember … at the table … brothers and sisters … My dad’s there … eat and laugh and talk and drink and enjoy with my family … There’s very few feelings like that in the world and a person can experience that through food* (Godderis, 2006a, p.255).

Echoing this, Ugelvik (2011) suggests that food gives individuals the “chance to figuratively climb the prison wall; it connects him to the world outside and reminds him of the life he is still a part of … he is positioned as a family member [for whom] the world outside … still exists” (Ugelvik, 2011, p.58).

## Discussion

Our meta-ethnography synthesises first-hand accounts about prison food reported in 17 studies (27 papers) globally to build understanding about people’s lived experience of prison and better inform those who seek to enhance the prison environment and improve the health and wellbeing of people in custody. The line of argument synthesis we present argues that experiences around food in prison, where choice and control are limited, affect prisoners’ lived experience of prison and their perceptions of status, identity, and social relationships. Despite the heterogeneity in the types of prisons which were the sites for the included studies, we highlight some striking similarities in prisoners’ experiences of prison food depending on whether they had autonomy over their food choices.

Prior studies have noted that prison-issued meals in some countries (England, Australia) can be considered nutritionally adequate (Edwards et al., 2009; Williams et al., 2009), in other countries (US, Poland, Spain) reports suggest that prison-issued diets are high in fat and sugar, low in fibre and contain few fresh vegetables or fruit (Soble et al., 2020; Stanikowski et al., 2020; Varoucha-Azcarate, 2019). Previous overviews of the pragmatic aspects of prison food, such as nutritional content and logistics around its production (Smoyer, 2019; Smoyer & Kjaer Minke, 2015) have concluded that people in custody struggle to maintain a balanced diet, especially in developing countries. Our synthesis demonstrates that across *all* countries studies, the lived experience for most in custody is of poor-quality, prison-issued meals, and in most countries outside Europe, prisoners also report inadequate portion size. The research in our synthesis also highlights that the location and timing of mealtimes were at odds with social and cultural norms outside prison. Thus, our findings agree with the assertion that “the quantity and quality of the food provided is insufficient, and the conditions in which it is served and eaten undermine respect for prisoners' dignity” (H.M.I.P., 2016, p.13).

Prison regimes, by necessity, remove choice and control from individuals and impose cycles of repetition that can cause anger and frustration (Woodall et al., 2014). Smith (2002) and Godderis (2006a) suggest that the removal of simple routine choices around everyday activities (i.e., what, when and how to eat) is a constant reminder of the lack of agency individuals living in prison have over their lives. The settings-based approach to health promotion is underpinned by core values such as participation, equity, empowerment and agency, respect and decency (Baybutt et al., 2014; Dooris et al., 2014; WHO, 1986). However, food in prison has been described as a largely a top-down expression of power with those in custody having little control or choice over what they eat (Smoyer, 2019). Despite these constraints, our synthesis highlights the ways in which, across all countries represented, those in custody find ways to exercise agency in an environment traditionally antithetical to empowerment and participation (Smith 2002; Woodall et al., 2014). Thus, beyond providing adequate nutrition, food carries clear symbolic meanings in prison. Consistent with other reviews, our findings suggest that through everyday food activities in prison, especially cooking, empowerment, participation, agency and identity can be negotiated and performed (Smoyer and Reeves 2015, 2019; Smoyer & Kjaer Minke, 2015).

The literature in our synthesis demonstrates that food is a powerful tool for creating and maintaining relationships. Those narratives which focus on self-cook facilities and the social groups formed around cooking and eating demonstrate the benefits of peer support to prisoners’ sense of wellbeing. Given that existing research on peer support in prisons suggests that it can lead to improved health, wellbeing, self-esteem, and confidence (Collica, 2010; South et al., 2014), understanding what role food can play in building and maintaining positive social relations is of tremendous value. Narratives in our synthesis also demonstrate that illicit cooking, what has been referred to as food-based resistance (Smoyer, 2019), characterised by rejecting or repurposing of prison-issued meals, is an opportunity for enacting individual agency. Our findings resonate with literature that suggests that cooking (with others or alone) can reduce anxiety and depression and increase feelings of self-efficacy and resilience especially in psychologically and socially disadvantaged populations (Farmer et al., 2018).

Most of those in custody are expected to return to the community at some point (WHO, (2019). It has already been noted that this represents an opportunity to tackle health problems in a way that impacts positively on individuals and the communities they return to (Enggist et al., 2014; Stürup—Toft et al., 2018). Woodall et al., (2014) point to a paradox that highlights the fact that reintegration requires those in custody to exercise some agency, control and choice but many experiences in prison constrain these. In their study of the significance of self-care skills to reintegration after imprisonment, Maruca et al., (2021) note that cooking for oneself and eating well were regarded by prisoners as one of the most important aspects of self-care on release. Rather than simply focus on prison food as good nutrition for an underserved population, the findings of our synthesis suggest that integrating cooking and sharing food into the everyday routine of prison life (Dooris, 2009) can “strengthen the resources available to people and empower[s] them to increase control over the determinants of health and to thrive” (Dooris et al., 2014, p.13).

### Implications for future research and practice

Providing opportunities to cook and share food while in custody, has the potential to enhance the prison environment, improve the health and wellbeing of those in prison, create and sustain supportive relationships and allow individuals an opportunity to enact their familial and cultural identity. Alienating those in prison from control and choice around their own consumptive choices seems to lead to risky behaviours either through illicit cooking and/or the consumption of ‘junk’ food. The research in our synthesis suggests that endorsing cooking and sharing food among those in custody can increase the legitimacy of the prison regime and decrease rule-breaking and resentment. The research in our review highlights the role of cooking and sharing food in constructing positive identities and relationships, with several examples of people working together, supporting each other and sharing resources. Food, beyond nutrition, carries meaning in society that create and maintain culture, relationships and identity and our synthesis demonstrates that food in prison is no exception and establishments should reflect this.

This synthesis also highlights gaps in knowledge. There is a need to understand the role food plays in family visits in order to appreciate how this aspect of the prison environment might be enhanced. The current literature base around family visits and how food is used to sustain family relationships is small (Comfort, 2002; Moran, 2013) but powerfully demonstrates that as a means of showing care and support, food is an important tool in sustaining familial and social relationships which can, in turn be crucial to individuals while in prison and support reintegration on release (Venema et al., 2022).

Reviews of prison food systems (Smoyer, 2019; Smoyer & Kjaer Minke, 2015) do not include the views and experiences of prison staff as they attempt to balance challenges around resources and logistics with the health and wellbeing of prison populations. Research that focuses on staff perceptions and experiences of prison issued food could offer additional perspectives and inform positive structural changes to the prison environment and interventions that focus on ways in which improve health and wellbeing among both staff and those in custody.

Meta-ethnography has been widely applied in the health-care sector to assess patients’ experiences of illness or interventions but we have not identified any prior example that explores experiences of people in custody. With its emphasis on connections made across qualitative data rather than simply aggregating instances, meta-ethnography is a robust and systematic method that could prove useful to prison researchers who seek to synthesise the everyday lived experiences of those in custody in the future.

### Limitations

The literature included in our synthesis may not be exhaustive. Although meta-ethnography is widely used in healthcare research, there is a lack of clarity surrounding each of the seven phases. We have provided descriptions of how we interpreted this method but others may have approached any one of the stages differently.

## Conclusions

This synthesis has explored the central role of food to the lived experience of people in custody. The potential of food to enhance the prison environment and support improvements in prisoner health and wellbeing is limited when the nutritional content is inadequate and/or the environment where food is served and eaten impacts negatively on human dignity. Providing opportunities for those in custody to cook and share food that better reflects familial and cultural identity can improve relationships, increase self-esteem, build and maintain life skills needed for reintegration, and express the values which underpin the setting-based approach to health promotion.

## Data Availability

The datasets created and analysed during the current study available from the corresponding author on reasonable request.
